# Downregulation of AKT3 Increases Migration and Metastasis in Triple Negative Breast Cancer Cells by Upregulating S100A4

**DOI:** 10.1371/journal.pone.0146370

**Published:** 2016-01-07

**Authors:** Astrid Grottke, Florian Ewald, Tobias Lange, Dominik Nörz, Christiane Herzberger, Johanna Bach, Nicole Grabinski, Lareen Gräser, Frank Höppner, Björn Nashan, Udo Schumacher, Manfred Jücker

**Affiliations:** 1 Center for Experimental Medicine, Institute of Biochemistry and Signal Transduction, University Medical Center Hamburg-Eppendorf, Martinistrasse 52, 20246 Hamburg, Germany; 2 Department of Hepatobiliary and Transplant Surgery, University Medical Center Hamburg-Eppendorf, Martinistrasse 52, 20246 Hamburg, Germany; 3 Center for Experimental Medicine, Department of Anatomy and Experimental Morphology, University Cancer Center, University Medical Center Hamburg-Eppendorf, Martinistrasse 52, 20246 Hamburg, Germany; Witten/ Herdecke University, GERMANY

## Abstract

**Background:**

Treatment of breast cancer patients with distant metastases represents one of the biggest challenges in today’s gynecological oncology. Therefore, a better understanding of mechanisms promoting the development of metastases is of paramount importance. The serine/threonine kinase AKT was shown to drive cancer progression and metastasis. However, there is emerging data that single AKT isoforms (i.e. AKT1, AKT2 and AKT3) have different or even opposing functions in the regulation of cancer cell migration *in vitro*, giving rise to the hypothesis that inhibition of distinct AKT isoforms might have undesirable effects on cancer dissemination *in vivo*.

**Methods:**

The triple negative breast cancer cell line MDA-MB-231 was used to investigate the functional roles of AKT in migration and metastasis. AKT single and double knockdown cells were generated using isoform specific shRNAs. Migration was analyzed using live cell imaging, chemotaxis and transwell assays. The metastatic potential of AKT isoform knockdown cells was evaluated in a subcutaneous xenograft mouse model *in vivo*.

**Results:**

Depletion of AKT3, but not AKT1 or AKT2, resulted in increased migration *in vitro*. This effect was even more prominent in AKT2,3 double knockdown cells. Furthermore, combined downregulation of AKT2 and AKT3, as well as AKT1 and AKT3 significantly increased metastasis formation *in vivo*. Screening for promigratory proteins revealed that downregulation of AKT3 increases the expression of S100A4 protein. In accordance, depletion of S100A4 by siRNA approach reverses the increased migration induced by knockdown of AKT3.

**Conclusions:**

We demonstrated that knockdown of AKT3 can increase the metastatic potential of triple negative breast cancer cells. Therefore, our results provide a rationale for the development of AKT isoform specific inhibitors.

## Introduction

Breast cancer is the most common malignancy in women and the third most frequent cancer worldwide [1]. Survival rates have continually improved over the last decades, mainly due to improved diagnosis and treatment of patients in an early, curable and non-metastatic state [2]. Therefore, morbidity and mortality are mainly determined by the occurrence of distant metastases, most frequently affecting bones, lungs, liver and brain [3,4]. The migration of tumor cells from the primary tumor to other organs is a complex, multi-step process. To establish distant metastasis, cancer cells must invade the surrounding tissue, intravasate into blood or lymph vessels, and finally extravasate and proliferate at a distant organ site [5–7]. However, little is known about the molecular mechanisms regulating this complex process. Thus, a better understanding of the metastatic process is essential to develop new diagnostic and therapeutic strategies.

The serine/threonine kinase AKT, a major downstream mediator of the phosphoinositide 3-kinase (PI3K)-pathway, was shown to regulate cancer progression and determine the metastatic potential of breast cancer cells by promoting migration and invasion, e.g. by upregulation of integrin β1 and matrix metalloproteinase (MMP) expression [8,9]. Activation of the PI3K/AKT signaling pathway is observed in up to 81% of breast cancer patients, as determined by immunohistochemical staining for AKT phosphorylated at serine residue 473 (pAKT S473) [10]. This reflects the frequent activation of receptor tyrosine kinases, activating mutations in PI3KCA and AKT1, as well as overexpression of AKT2 or AKT3 and inactivation of the tumor suppressor gene PTEN in breast cancer [11,12]. Activation of AKT was shown to correlate with decreased disease free survival [13] and to be associated with tumor progression [14,15]. However, emerging data indicates that the three highly homologous AKT isoforms (i.e. AKT1, AKT2 and AKT3) may play different or even functional opposing roles in the regulation of migration and invasion [8,9,16]. For example, overexpression of AKT1 was shown to inhibit motility, whereas overexpression of AKT2 promotes motility and migration in breast cancer cells, the latter resulting in increased formation of metastases [17–19]. However, the functional role of AKT3 is least examined and therefore poorly understood. Recently, AKT3 was shown to be frequently overexpressed in triple negative breast cancer (TNBC), suggesting that AKT3 might drive the progression of triple-negative breast cancer [20]. On the other hand, recent data indicates that AKT3 inhibits cancer cell migration, which may therefore influence the metastatic potential of tumor cells. Chung et al. demonstrated that overexpression of N-cadherin decreases the expression of AKT3, resulting in increased motility of breast cancer cells [21]. In addition, Phung et al. showed that knockdown of AKT3 decreases the expression of Rictor in vascular tumor cells, resulting in increased p70S6 kinase activity, thereby increasing migration [22]. However, the molecular mechanisms and effectors downstream of AKT3 in the regulation of cancer cell migration and metastasis remain to be elucidated.

In this study, we provide first evidence that downregulation of AKT3 in TNBC cells increases migration and the formation of lung metastasis by upregulating S100A4. S100A4 expression has been implicated to the metastatic potential of cancer cells and a correlation between the expression of S100A4 and the metastatic state and prognosis was shown for several cancer entities [23–26].

## Material and Methods

### Chemicals and reagents

Antibodies against pan AKT, AKT1, AKT2, pAKT (S473), pAKT (T308), pGSK3β (S9), pmTOR (S2448), pERK (T202/Y204), Rictor, and pS6 (S240/244) were purchased from Cell Signaling Technology (Danvers, MA). Antibodies against ERK1/2 and HSC-70 were purchased from Santa Cruz. AKT3 antibody was obtained from Millipore (Schwallbach, Germany). Antibody against S100A4 was purchased from Dako (Glostrup, Denmark).

### Cell culture and Proliferation

MDA-MB-231 (HTB-26) cells were obtained from the American Type and Culture Collection (ATCC) (Rockville, MD, USA). Hep3B cells were a kind gift from Prof. Dr. H. Will at the Heinrich Pette Institute, Hamburg, Germany. Both cell lines were maintained in DMEM, supplemented with 10% (v/v) FCS, and 1% (v/v) penicillin and streptomycin. BT549 cells were a kind gift of Dr. Harriet Wikman-Kocher at the Department of Tumor Biology, UKE. Cells were maintained in RPMI, supplemented with 10% (v/v) FCS, and 1% (v/v) penicillin and streptomycin. Cells were cultured at 37°C in a humidified atmosphere containing 5% CO_2_. All cell lines were used at low passage number not exceeding 20 passages. Proliferation of cells was analyzed by manual cell counting over four consecutive days, performed in triplicates. The experiment was repeated at least once.

### Western blot analysis

Western blot analysis was performed as described previously [27]. Protein expression was quantified using LAS-3000 Imager and AIDA Image Analyzer v.3.44 from Fuji software (Raytest, Straubenhardt, Germany).

### Lentiviral knockdown of AKT isoforms

pLKO.1-puro vector encoding either shRNAs directed against AKT1, AKT2, AKT3 or scrambled shRNA were purchased from Sigma-Aldrich (Taufkirchen, Germany). Generation of pseudotyped lentiviruses and transduction were performed as described previously [27]. Briefly, cells were transduced with an AKT isoform shRNA containing vector and selected by addition of puromycin (Sigma-Aldrich, Taufkirchen, Germany) to culture medium at a final concentration of 1.5 μg/ml for at least one week. Then, cells were sequentially transduced with a vector containing a second AKT isoform-specific shRNA followed by selection with culture medium containing puromycin and neomycin (final concentration 800 μg/ml). Controls were transduced sequentially with two non-target control vectors.

### RNA interference of S100A4

MDA-MB-231 cells were transfected with S100A4 siRNA (25pmol per reaction, Dharmacon, GE healthcare, life sciences) or non-targeting siRNA as negative control using Lipofectamine 2000 (Invitrogen) for 48 hours. Knockdown was confirmed by Western blot analysis.

### Transwell assay, scratch assay and live cell imaging

Transwell migration assays were performed as described before [27]. Briefly, 3x10^5 cells in serum free medium were added to the upper chamber of transwell filters (8μm pore size; Corning Cat. #3422) in triplicates. Medium containing 10% FCS was added to the lower chambers. After 16 hours of incubation, cells on the lower side of the insert were labeled with Calcein-AM, according to the instructions provided by the manufacturer, and fluorescence signal was detected using a M200 Infinite plate reader (Tecan, Switzerland).

For scratch assays, cells were seeded in 12 or 24 well tissue culture plates (Greiner bio-one, Cellstar) and grown to about 90% confluence. The cell monolayer was scraped with a 200μl pipette tip and culture medium was replaced by fresh medium to remove detached cells. The 12 well plates were placed in a Zeiss Axiovert 200M equipped with an environmental chamber maintained at 37°C and 5% CO2 humidified atmosphere during imaging. Time lapse images with an intervall of five minutes of at least three different locations per scratch were recorded using Volocity 6.1.1 3D Image Analysis software (PerkinElmer). Velocity of migration was analyzed by using the software Volocity 6.1.1 3D Image Analysis. Each experiment was performed at least twice.

### Chemotaxis Assay

The experiment was carried out using IBIDI^TM^ 3D-Chemotaxis slides for *in vitro* chemotaxis measurement [28]. FCS with a final concentration of 10% (v/v) was used as a chemoattractant. Cells were seeded at high density (3x10^6/ml) into the observation area of the 3D chemotaxis slides, and reservoirs were filled according to the instructions provided by the manufacturer. Time lapse images were recorded as described above. Velocity and euclidean distance were analyzed using ImageJ software.

### Subcutaneous tumor xenograft model and *in vivo* analysis

This study was carried out in strict accordance with the recommendations in the Guide for the Care and Use of Laboratory Animals of the National Institutes of Health. All experimental protocols were approved by local authorities (Ministry of Health and Consumer Protection, Hamburg, Germany, Permit Number G11/12). 1x10^6 MDA-MB-231 cells were injected subcutaneously into SCID mice (female, age six weeks, n = 8 to 10 per group, obtained from Charles River, Sulzfeld, Germany). Tumor growth was monitored regularly, and mice were withdrawn from the experiment when an abortion criterion was met (tumor size > 1.5 cm, loss of body weight, poor general condition). Upon necropsy, xenograft primary tumors as well as blood, lung tissue and bone marrow were harvested. A portion of each tumor was fixed with 10% formalin or snap frozen on liquid nitrogen for Western Blot analysis.

### Quantification of disseminated tumor cells by Alu-PCR

The quantification of disseminated tumor cells by Alu-PCR was performed as described previously [29]. DNA concentrations of all samples were quantified using a NanoDrop spectrophotometer (Peqlab). As the content of detectable Alu-sequences in the following qPCR would have been affected simply by varying DNA concentrations, all lung and bone marrow DNA samples were normalized to 30 ng/mL using AE buffer (Qiagen). Quantitative PCR (qPCR) was performed with established human-specific Alu primers [29]. Two microliters total DNA (i.e., 60 ng lung-DNA) were used for each qPCR. Numerical data were determined against a standard curve. The detection limit for specific human Alu sequence signals was determined for each tissue type by testing DNA from five healthy (noninjected) SCID mice of similar sex and age. For each sample, analyses were performed in duplicates and as independent experiments at least twice.

### Immunohistological staining of tumor samples

IHC was performed with polyclonal rabbit anti-S100A4 (Dako) on 4 μm xenograft tumor sections. Rabbit negative control (Dako) served as isotype control. Briefly, sections were dewaxed and antigen retrieval was carried out in citrate buffer (pH 6) at 60°C overnight. Non-specific binding was blocked by incubating the sections in 10% normal swine serum followed by incubation with the primary antibody (1:10 in antibody diluent, Dako). After washing, sections were incubated with biotinylated swine-anti-rabbit antibody. Detection of S100A4 antibody was performed by the streptavidin-alkaline phosphatase kit (ABC-AP; Vector Labs.). Enzyme reactivity of the AP complex was visualized using DAKO liquid permanent red.

### RNA isolation, cDNA synthesis and qPCR

RNA isolation and cDNA synthesis were performed in triplicates as described previously [27]. Sense and antisense oligonucleotide primers for amplification of human mRNAs were designed based on published cDNA sequences (NCBI GenBank), (primer sequence S100A4: forward CTGATGAGCAACTTGGACAG, reverse CATCAAGCACGTGTCTGAAG; GAPDH: forward GAGTCAACGGATTTGGTCGT, reverse TTGATTTTGGAGGGATCTCG). Oligonucleotide primers were obtained from MWG (Ebersberg, Germany). qPCR was performed on the capillary-based LightCycler (Roche, Grenzach, Germany) using the Sybr Premix Ex™ II (Tli RNaseH Plus) Kit (TaKaRa Bio Inc., Japan), and calculation of relative mRNA levels was based on the delta-delta cycle threshold method as described previously [27]. Gene expressions were normalized to the reference gene glyceraldehyde 3-phosphate dehydrogenase (GAPDH).

### Statistical Analysis

Data analysis was performed with Microsoft Excel and GraphPad Prism. Student’s t-Test (unpaired, 2-tailed) or Kruskal-Wallis test were calculated based on the data of at least three independent experiments. Bonferroni correction for multiple testing was performed where applicable. Results were considered significant if p<0.05. All error bars represent SD, unless indicated otherwise.

## Results

### Knockdown of AKT3 leads to increased migration in MDA-MB-231 cells

**Fig 1:**
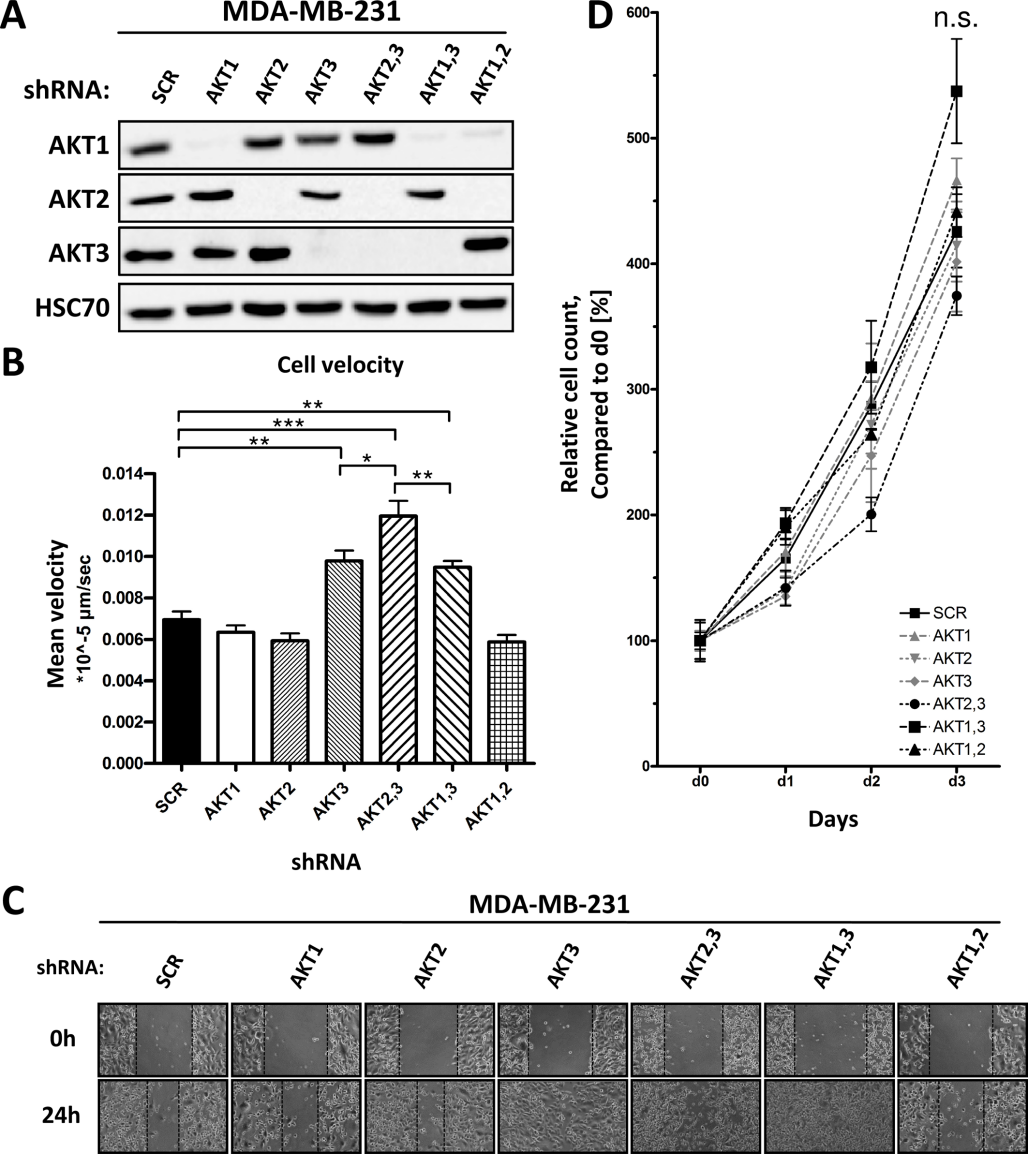
Effect of AKT isoform single and double knockdown on migration and proliferation. (A) Knockdowns of AKT isoforms in MDA-MB-231 cells were performed by lentiviral transduction using AKT isoform specific shRNAs. Efficiency was confirmed by Western blot analysis. (B)-(C) Analysis of cell migration using scratch assay technique. A confluent monolayer was scratched using a 200μl pipette tip and cell migration was analyzed using time lapse video microscopy. Mean single cell velocity (B) of MDA-MB-231 control and AKT isoform knockdown cells. One experiment out of three is shown (Bars: SD. *, p < 0,05, **, p < 0,01. ***, p < 0,001). (C) Representative image of a scratch assay after 0 and 24 hours, the dotted line indicates the leading front. (D) Proliferation was analyzed by manual cell counting over four consecutive days, performed in triplicates, proliferation is shown as relative cell count compared to day 0 (Bars: SD, n.s., p>0,05).

To investigate the functional role of each AKT isoform in the regulation of migration and metastasis, expression of either one or two AKT isoforms was downregulated in the TNBC cell line MDA-MB-231 using two independent sets of isoform-specific shRNAs. Until today, only few AKT substrates have been identified that are either preferred by or specific for single AKT isoforms [17], and the AKT isoforms are believed to show a significant functional overlap [30]. Therefore, double AKT isoform knockdown cells retaining only one AKT isoform were used to evaluate synergy or redundancy between two AKT isoforms. Downregulation of AKT isoforms was effective and stable over time, without affecting the expression of the remaining AKT isoforms (Fig 1A). The effect of AKT isoform knockdown on migration of MDA-MB-231 cells was analyzed by performing scratch assays using live cell imaging. We observed an increased migration of cells lacking AKT3 (AKT3 single knockdown as well as AKT2,3 and AKT1,3 double knockdown) compared to control cells, as indicated by an increased overall cell velocity (Fig 1B, representative time lapse videos can be found in S1, S2 and S3 Videos) and a faster wound healing rate (Fig 1C). Downregulation of AKT1 and AKT2, either alone or in combination, had no significant effect on migration. Similar results were obtained using an independent set of shRNAs, confirming that depletion of AKT3 increases migration (S1 Fig). To rule out that this effect was due to an increased proliferation, cells were seeded into 6 well plates and the cell number was counted on three consecutive days. Neither single nor double knockdown of any AKT isoform had a significant impact on proliferation compared to control cells (Fig 1D). To further extent our results, we analyzed the effect of single AKT isoform knockdown on migration in a second triple negative breast cancer cell line, BT549, as well as in the hepatocellular carcinoma cell line Hep3B. As shown in S1 and S2 Figs, only knockdown of AKT3 resulted in increased migration. Taken together, we have shown that depletion of AKT3 increases cell migration independent of cell proliferation.

### Downregulation of AKT3 increases transmigration and chemotaxis

**Fig 2:**
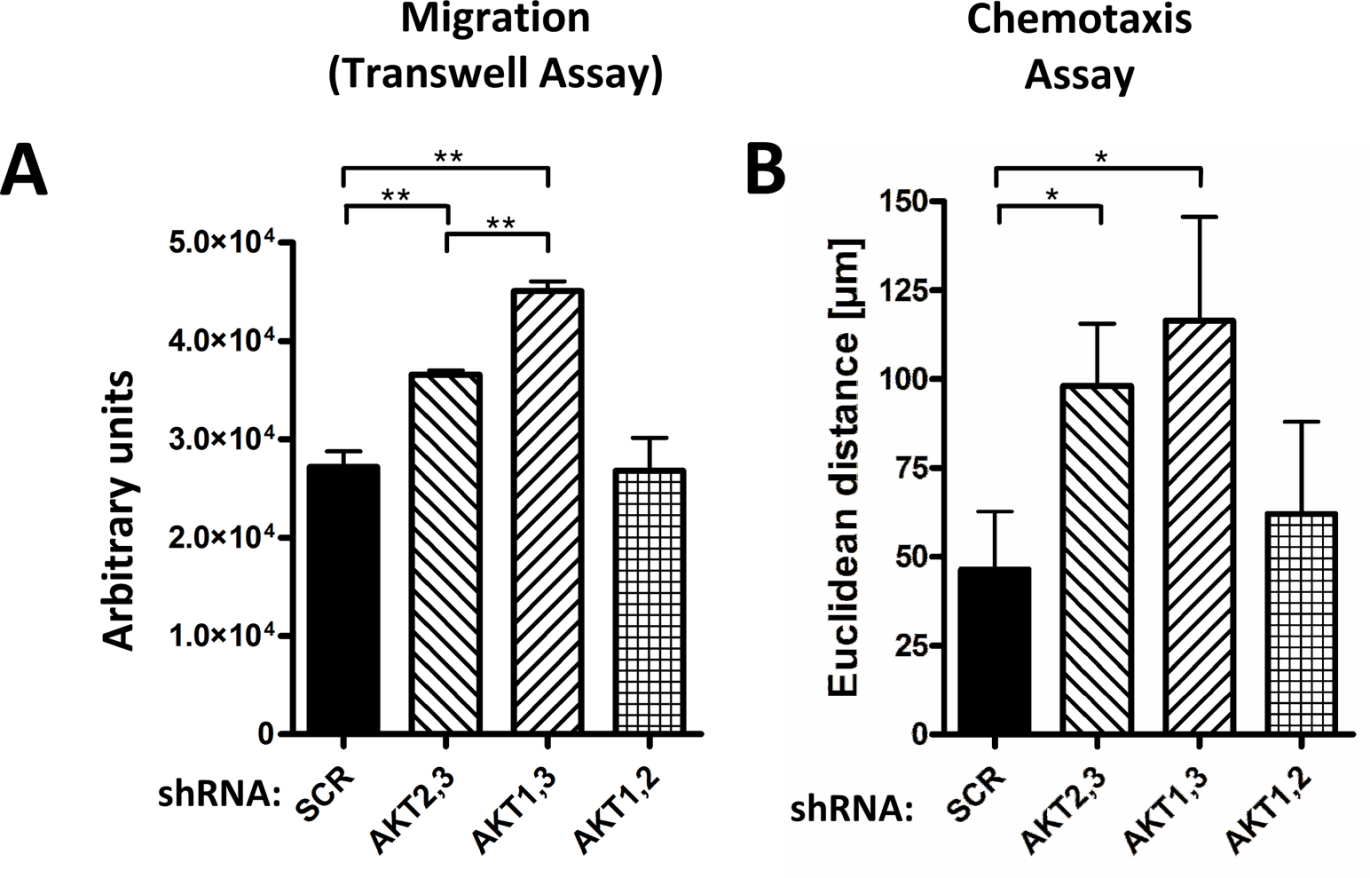
Depletion of AKT3 increases transmigration and chemotaxis in MDA-MB-231 cells. (A) For transwell assays, cells were seeded into the upper chamber of transwell filters and allowed to migrate towards a FCS gradient for 16 h. Cells that have reached the lower side of the transwell insert were stained with Calcein AM, and fluorescence signal was detected using a Tecan Infinite 200M reader (Bars: SD. **, p < 0,01). (B) For chemotaxis analysis, cells were seeded into the observation area of chemotaxis slides, and reservoirs were filled to generate a stable FCS gradient (final concentration of 10%). Time lapse images were recorded to analyze the effective chemotaxis (Bars: SD. *, p < 0,05).

We aimed to confirm these results using transwell migration assays for double AKT isoform-knockdown cells (Fig 2A). Downregulation of AKT2,3 and AKT1,3 resulted in an increased transmigration of cells compared to control and AKT1,2 knockdown cells. Of note, migration of AKT3 knockdown cells appeared less coordinated compared to control cells (S1–S3 Videos). To investigate whether downregulation of AKT3 impairs directed migration, chemotaxis assays were performed, measuring the effective migration (i.e. Euclidean distance per time interval) towards an FCS gradient. As shown in Fig 2B, AKT2,3 and AKT1,3 double knockdown cells showed an increased chemotaxis compared to AKT1,2 knockdown and control cells, indicating that depletion of AKT3 does not impair directed migration.

### AKT3 knockdown increases the expression of the promigratory protein S100A4

**Fig 3:**
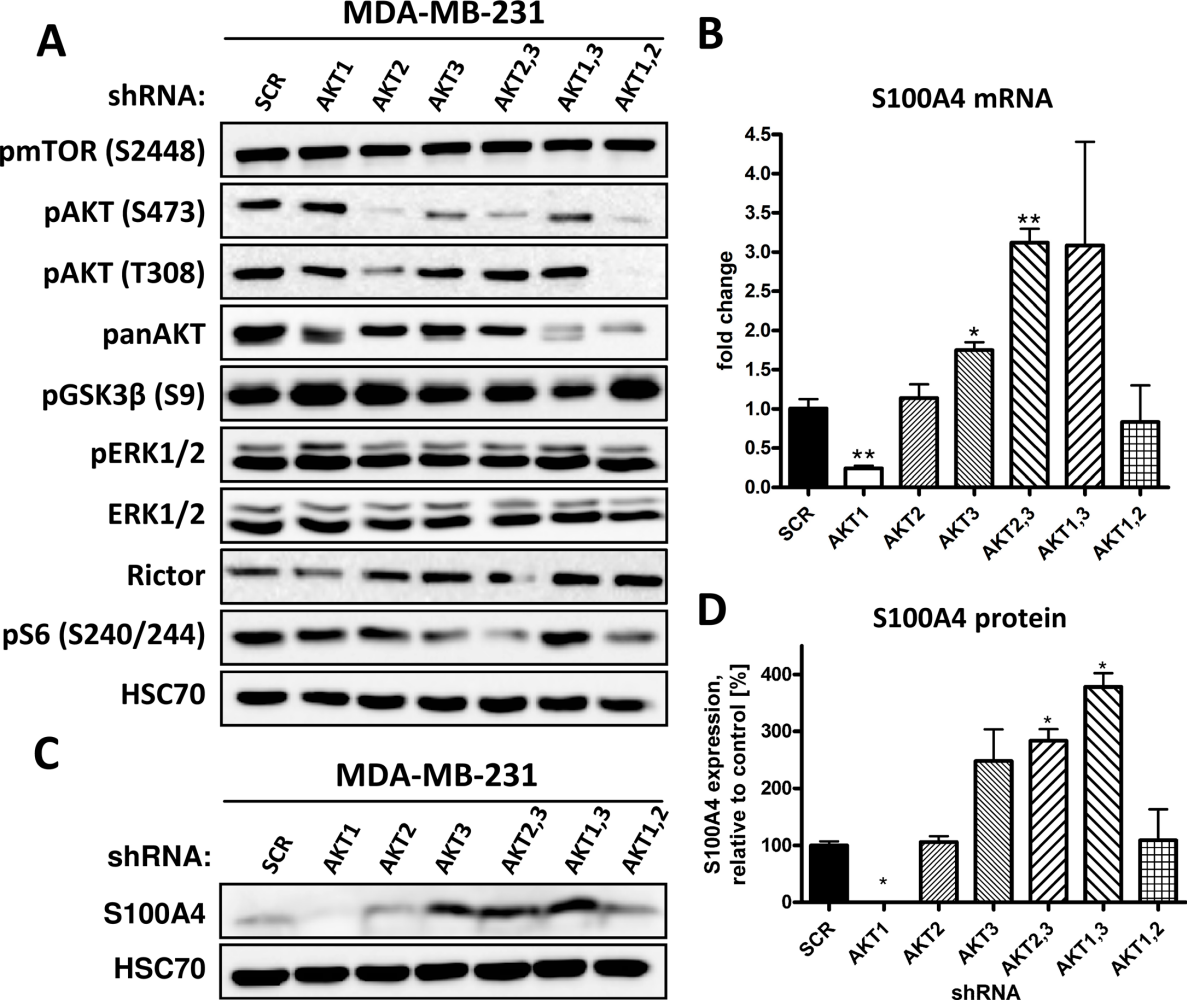
Knockdown of AKT3 increases the expression of S100A4 protein. (A) AKT signaling pathway activity after downregulation of AKT isoforms was analyzed by Western blot with antibodies directed against the indicated proteins. HSC70 was used as loading control. (B) Relative mRNA levels of S100A4 were measured by qPCR (Bars: SD. *, p < 0,05. **, p < 0,01). (C) Expression of S100A4 in MDA-MB-231 control and AKT isoform knockdown cells was analyzed by Western blot, HSC70 was used as loading control. (D) S100A4 expression was quantified from Western blot analysis of triplicates and data were normalized to corresponding HSC70 controls (Bars: SD. *, p < 0,05).

Next, the phosphorylation of AKT at serine residue 473 (S473) and threonine residue 308 (T308), reflecting the activation status of AKT, were analyzed. Downregulation of any two AKT isoforms resulted in a significant reduction of pAKT S473 (Fig 3A). Depletion of AKT1 increased and knockdown of AKT2 decreased overall levels of pAKT S473, whereas downregulation of AKT3 had no effect (Fig 3A).

To date, the molecular mechanisms promoting migration and invasion after inhibition or knockdown of AKT remain poorly understood. The expression and phosphorylation of AKT substrates, which were shown to regulate migration, were investigated by Western blot analysis (Fig 3A). Virtakoivu et al. have shown that integrin β1 activity is regulated by AKT1 and AKT2 in prostate cancer cells [31]. However, we could not detect changes in integrin β1 activity in MDA-MB-231 AKT isoform knockdown cells (data not shown). Furthermore, there was no significant change in the expression of Rictor after knockdown of any AKT isoform in MDA-MB-231 cells (Fig 3A), which had been shown to be regulated by AKT3 in vascular tumor cells [22]. Knockdown of AKT3 even resulted in decreased phosphorylation of downstream mTORC1 substrate S6 ribosomal protein at serine residue 240/244 (Fig 3A).

To further elucidate the underlying molecular mechanisms of an increased migration after knockdown of AKT3, the expression of several promigratory proteins was analyzed using qPCR (data not shown). We found a significant upregulation in the expression of S100A4 both at transcriptional and protein level in MDA-MB-231 cells lacking AKT3 (Fig 3B–3D). S100A4, a member of the S100 protein family, is involved in a broad range of biological functions as well as in the regulation of migration and invasion of cancer cells [25,26]. Overexpression of S100A4 was shown to promote motility, invasion and formation of metastasis in TNBC cell line MDA-MB-231 [32] as well as other cell lines [23]. Furthermore, there is a strong correlation between the expression of S100A4 and poor overall survival as well as the presence of distant metastases in multiple cancer entities, including breast cancer [23,24,26,33].

### Knockdown of S100A4 reverses the promigratory phenotype in AKT3 lacking cells

**Fig 4:**
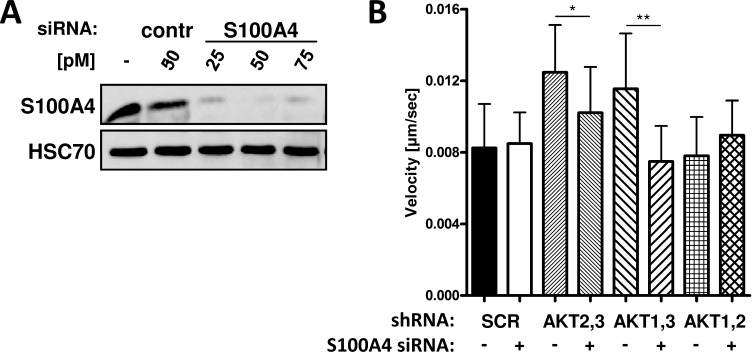
Knockdown of S100A4 reverses the promigratory effect in AKT3 lacking cells. MDA-MB-231 double knockdown cells were transfected with S100A4 siRNA or non-targeting siRNA as negative control. Knockdown was confirmed by Western blot analysis (A). The impact of S100A4 expression on migration was analyzed by scratch assay using time-lapse video microscopy. Mean single cell velocity is shown (Bars: SD. *, p < 0,05, **, p < 0,01) (B).

To analyze whether the increased migration in MDA-MB-231 AKT3 knockdown cells is mediated by an increased expression of S100A4, cells were transfected with a siRNA targeting S100A4 (Fig 4A). We observed a significant decrease in cell velocity after knockdown of S100A4 in AKT2,3 and AKT1,3 knockdown cells, whereas migration of control and AKT1,2 knockdown cells was not affected (Fig 4B). These findings indicate that knockdown of AKT3 increases migration via upregulation of S100A4 protein expression.

### Knockdown of AKT3 increases the metastatic potential of MDA-MB-231 cells

**Fig 5:**
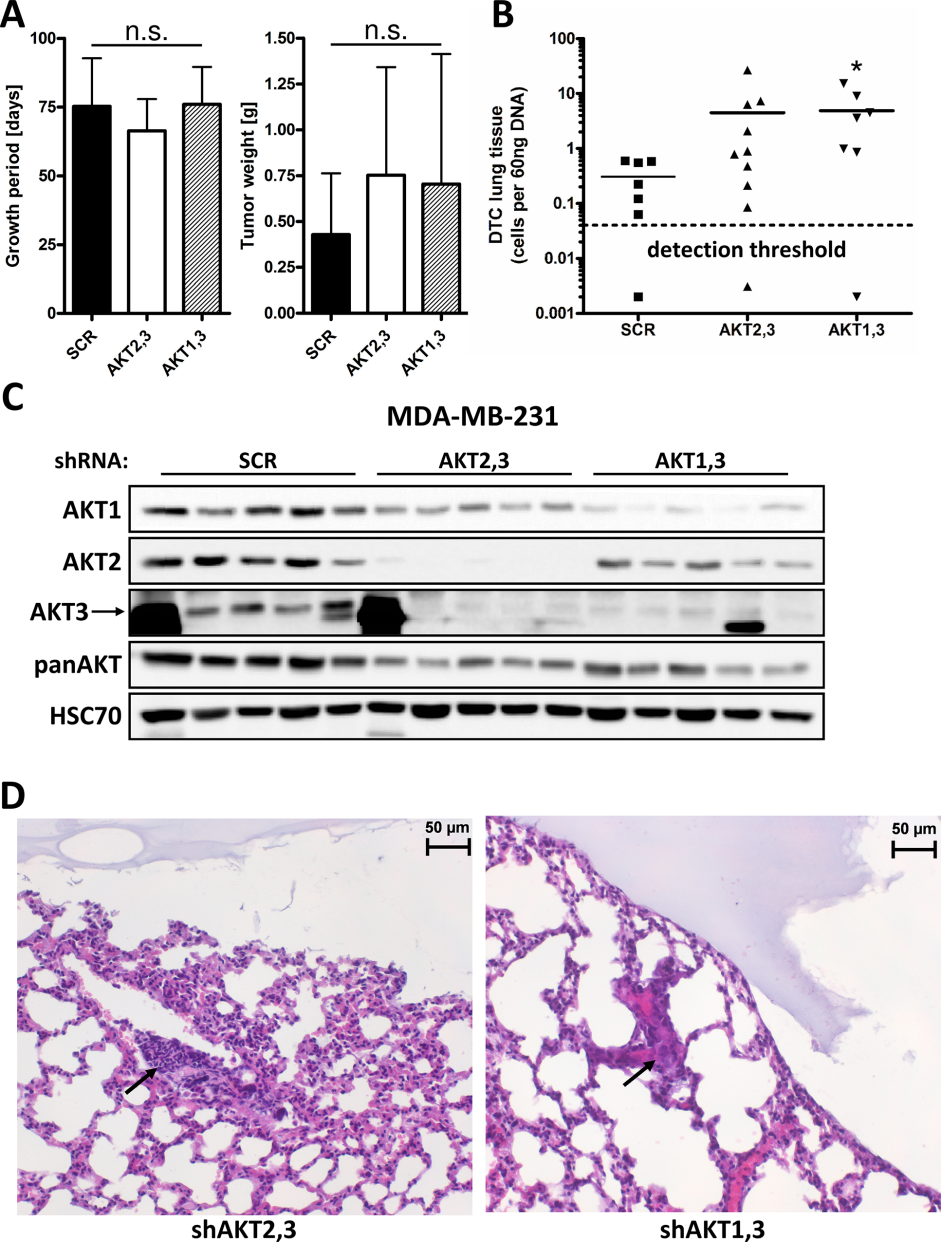
Knockdown of AKT3 increases the metastatic potential of MDA-MB-231 cells *in vivo*. AKT2,3 and AKT1,3 double knockdown cells as well as control cells were injected subcutaneously into SCID mice (8 to 10 mice per group). Tumors were allowed to grow until a termination criterion was met. (A) Growth period and tumor weight upon necropsy (Bars: SD, ns., p>0,05). (B) The number of disseminated tumor cells in the left lung of mice was quantified by Alu-PCR. (Bars: SD, *, p<0,05) (C) Protein lysates were prepared from 5 tumors per group, and AKT isoform knockdown were analyzed by Western blot. (D) Representative images from histological examination of the right lungs from mice injected with AKT2,3 and AKT1,3 knockdown cells showing macroscopically detectable metastases.

**Fig 6:**
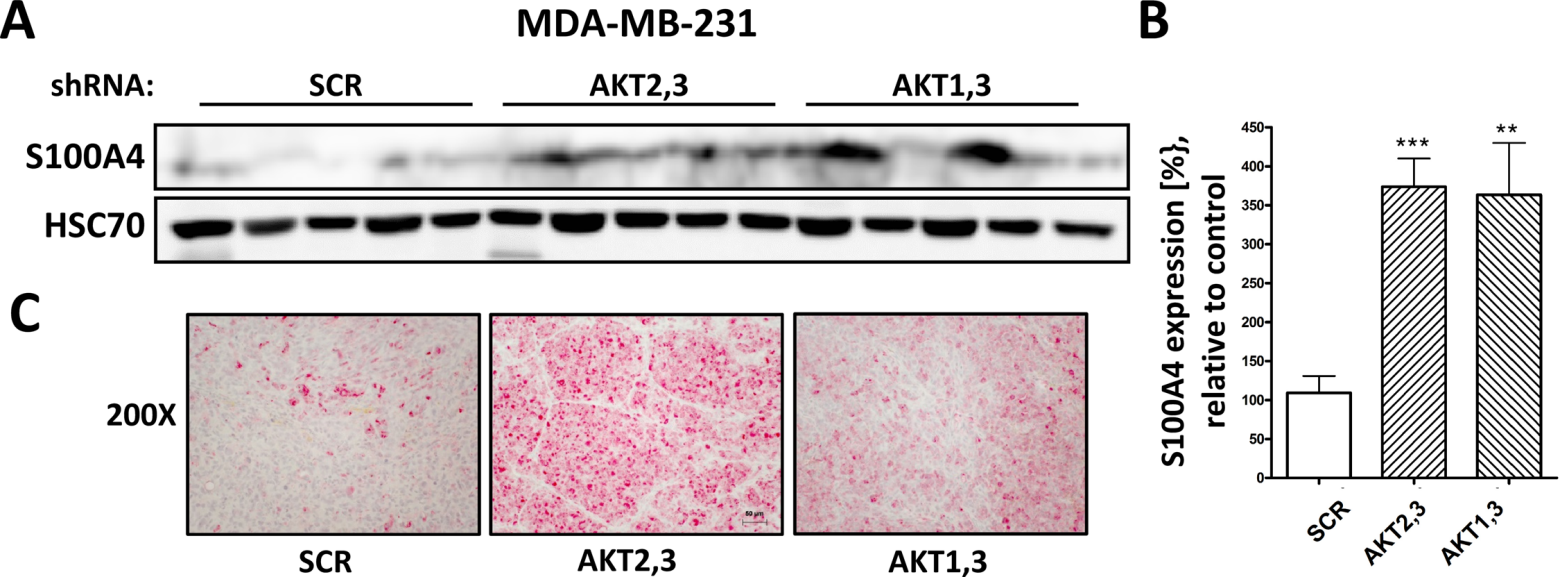
S100A4 is overexpressed in AKT3 lacking primary tumors. (A) Protein lysates were prepared from five primary tumors per group and expression of S100A4 was analyzed by Western blot analysis. (B) S100A4 expression was quantified from Western blot analysis of five primary tumor tissue samples. Data were normalized to corresponding HSC70 controls (Bars: SD. **, p < 0,01, ***, p < 0,001). (C) Immunohistochemical staining of S100A4 in xenograft tumor sections. One representative image is shown (magnification 200X).

To investigate the functional relevance of increased migration and S100A4 expression in AKT3 lacking cells *in vivo*, AKT2,3 and AKT1,3 double knockdown cells as well as controls were injected subcutaneously into SCID mice (8 to 10 mice per group). Since we detected no significant changes in migration after downregulation of AKT1 or AKT2, and since double knockdown of AKT2,3 seemed to increase migration even further compared to knockdown of AKT3 alone (Fig 1B), AKT3 double knockdown cells were used for *in vivo* experiments. Tumor take was observed in 88%, 88% and 100% of animals for SCR, AKT2,3 and AKT1,3 knockdown cells, respectively. Tumors were allowed to grow until a termination criterion was met according to local animal welfare regulations. There were no significant differences in the duration of growth period or tumor weight upon necropsy at the end of the experiment (Fig 5A). To analyze the metastatic burden, the number of disseminated tumor cells (DTCs) in the left lung of mice was quantified by Alu PCR (as described in Material and Methods). DTCs were detected in 86% (6 of 7), 86% (6 of 7) and 90% (9 of 10) of animals injected with SCR, AKT2,3 and AKT1,3 knockdown cells, respectively. The number of DTCs was significantly higher in the AKT1,3 knockdown condition compared to controls, and a borderline increase in DTCs was detected in lungs from mice in the AKT2,3 knockdown group (Fig 5B). There was no significant correlation between tumor weight and the number of DTCs in any group. Knockdown of AKT isoforms was confirmed by Western Blot in tumor tissue samples from five mice per group (Fig 5C). The presence of macroscopically detectable metastases was confirmed upon histological examination of the right lungs from mice injected with AKT2,3 and AKT1,3 knockdown cells (Fig 5D), whereas no metastases were found in the control group. The expression of S100A4 in primary tumor samples was analyzed by Western blot analysis, showing a significantly increased overall expression of S100A4 in tumors lacking AKT3 compared to controls (Fig 6A and 6B). These results were confirmed upon immunohistochemical staining of S100A4 in tissue samples from the primary tumors (Fig 6C).

## Discussion

In this study, we have demonstrated that knockdown of AKT3, but not AKT1 or AKT2, promotes migration and the metastatic potential in the TNBC cell line MDA-MB-231. Our results are in line with emerging data that the single AKT isoforms exert different or even opposing roles in the regulation of migration as well as other cellular functions [27,34,35]. For example, Phung et al. have shown that knockdown of AKT3 increases migration of vascular tumor cells by reducing the expression of Rictor, resulting in higher S6-kinase activity [22]. In addition, knockdown of AKT1 or AKT2, but not AKT3, facilitated cell migration via an increase in activity of integrin β1 in prostate cancer cells [31]. However, we detected no upregulation of S6-kinase activity, nor significant changes in the expression of Rictor or activation of integrin β1 in MDA-MB-231 cells after knockdown of AKT3 or any other AKT isoform. Chung et al. demonstrated that overexpression of N-cadherin leads to increased migration by reducing the expression of AKT3 in breast cancer cells [21]. Since MDA-MB-231 cells express no or very low levels of N-cadherin [36,37], we were unable to detect changes in the expression of N-cadherin after downregulation of AKT3 (data not shown). Instead, we have demonstrated that the increased migration after knockdown of AKT3 is mediated by an increased expression of S100A4 calcium-binding protein. The role of S100A4 in migration, invasion and metastasis as well as the impact of S100A4 expression levels on the overall prognosis of cancer patients have been documented in a broad range of different cancer entities [25,26,32,38,39]. S100A4 was shown to be involved in epithelial to mesenchymal transition and to mediate migration and metastasis by upregulating matrix metalloproteinases [32,33] as well as through the regulation of Src-FAK signaling [26]. However, the underlying molecular mechanisms mediating an increased expression of S100A4 after depletion of AKT3 remain to be elucidated. S100A4 was shown to be regulated mainly at transcriptional and mRNA level, i.e. by altering the methylation status of CpG islands in the S100A4 gene promotor regions, nuclear factor of activated T-cells 5 (NFAT5) activity and indirectly via expression of several miRNAs [40]. NFAT5 is involved in a variety of physiological cellular functions including regulation of hypertonic stress and cardiac development [41]. However, recent data also provide evidence that NFAT5 plays an important role in cancer progression by promoting the formation of metastasis [38,40,42]. NFAT5 expression was associated with poor prognosis in several cancers entities, including renal carcinoma and breast cancer [38,40,42]. Li et al recently demonstrated that NFAT5 promotes the expression of S100A4 in breast cancer cells [43] and downregulation of NFAT5 decreases S100A4 expression. Since PI3K and AKT1 were shown to be involved in the control of NFAT5 activity [44], AKT3 might regulate S100A4 expression via NFAT5. Furthermore, both, NFAT5 and S100A4 are regulated by microRNAs [45], including miRNA-21, miRNA-155, members of the miRNA200 family and miRNA-568 [33,43,46]. MicroRNAs (miRNA) are small noncoding RNAs that control the expression of genes by posttranscriptional modification, either by inhibiting protein translation or direct targeting of mRNAs [47–49]. AKT was shown to regulate various miRNAs in an isoform specific way [16,50]. Therefore, further experiments will be necessary to uncover the mechanisms involved in the regulation of S100A4 expression after knockdown of AKT3.

In summary, we have demonstrated that knockdown of AKT3 regulates migration in TNBC breast cancer cells via upregulation of S100A4. Recent studies have highlighted that the different AKT isoforms may promote but also inhibit migration and metastasis, possibly depending on the exact cellular context. Although panAKT inhibitors are already being tested in clinical trials [51,52], a better understanding of the distinct roles of AKT isoforms highlights the need for the development of isoform specific inhibitors [53]. In light of the growing evidence that AKT3 inhibits cancer cell migration [21] and the fact that AKT isoforms are differentially expressed, with AKT1 being the most abundant AKT isoform in the majority of most breast cancer cell lines [20], inhibitors that preferably target AKT1 and AKT2 might be a promising therapeutic approach.

## Supporting Information

S1 Fig Knockdown of AKT3 increases migration in MDA-MB-231 and BT549 cells. (A) Knockdowns in MDA-MB-231 cells were performed by lentiviral transduction using a second set of independent AKT isoform specific shRNAs (#2). Efficiency was confirmed by Western blot analysis. (B) Analysis of migration of cells lacking AKT3 using scratch assay technique. A confluent monolayer was scratched using a 200μl pipette tip and cell migration was analyzed using time lapse video microscopy. The mean cell velocity of MDA-MB-231 control and AKT3 knockdown cells is shown. (Bars: SD. ***, p < 0,001). (C) AKT isoform specific knockdowns in BT549 cells were generated by lentiviral transduction using AKT isoform specific shRNAs as described in section 3.6. Knockdown efficacy was confirmed by Western blot analysis. (D) Migration of AKT isoform knockdown cells was analyzed by scratch assay and live cell imaging techniques, as described in section 3.6. The mean cell velocity of BT549 control and AKT3 knockdown cells is shown. (Bars: SD. ***, p < 0,001).
(TIF)

S2 Fig Effect of AKT isoform single knockdown on migration and proliferation of Hep3B cancer cells. (A) AKT isoform specific knockdowns in Hep3B cells were generated by lentiviral transduction using AKT isoform specific shRNAs. Knockdown efficacy was confirmed by Western blot analysis. (B)-(C) Analysis of cell migration using scratch assay technique. A confluent monolayer was scratched using a 200μl pipette tip and cell migration was analyzed using time lapse video microscopy, as described in section 3.6. Mean single cell velocity of Hep3B control and AKT isoform knockdown cells is given in (B). One representative experiment out of three is shown (Bars: SD. **, p < 0,01. ***, p < 0,001). (C) Representative images of the scratch assay after 0, 12, 24 and 36 hours are shown. (D) Proliferation was analyzed by manual cell counting over four consecutive days, performed in triplicates. Proliferation is shown as relative cell count normalized to day 0 (Bars: SD, n.s., p>0,05).
(TIF)

S1 Video Time lapse video of migrating SCR controls vs. AKT2,3 double knockdown MDA-MB-231 cells. Representative live cell imaging video of migrating MDA-MB-231 double knockdown cells. A confluent monolayer was scratched using a 200μl pipette tip and cell migration was analyzed using time lapse video microscopy. Pictures were taken every five minutes to generate videos.
(MOV)

S2 Video Time lapse video of migrating SCR controls vs. AKT1,3 double knockdown MDA-MB-231 cells. Representative live cell imaging video of migrating MDA-MB-231 double knockdown cells. A confluent monolayer was scratched using a 200μl pipette tip and cell migration was analyzed using time lapse video microscopy. Pictures were taken every five minutes to generate videos.
(MOV)

S3 Video Time lapse video of migrating SCR controls vs. AKT1,2 double knockdown MDA-MB-231 cells. Representative live cell imaging video of migrating MDA-MB-231 double knockdown cells. A confluent monolayer was scratched using a 200μl pipette tip and cell migration was analyzed using time lapse video microscopy. Pictures were taken every five minutes to generate videos.
(MOV)

## Abbreviations

PI3K: phosphoinositide 3-kinase
PI3KCA: catalytic subunit of phosphoinositide 3-kinase
TNBC: triple negative breast cancer
DTC: disseminated tumor cells
PBS: phosphate buffered saline
