# Supplementary figures and images for: Downregulation of AKT3 Increases Migration and Metastasis in Triple Negative Breast Cancer Cells by Upregulating S100A4
Source: PLoS One. 2016 Jan 7;11(1):e0146370. doi: 10.1371/journal.pone.0146370 (PMC4704820; doi:10.1371/journal.pone.0146370)

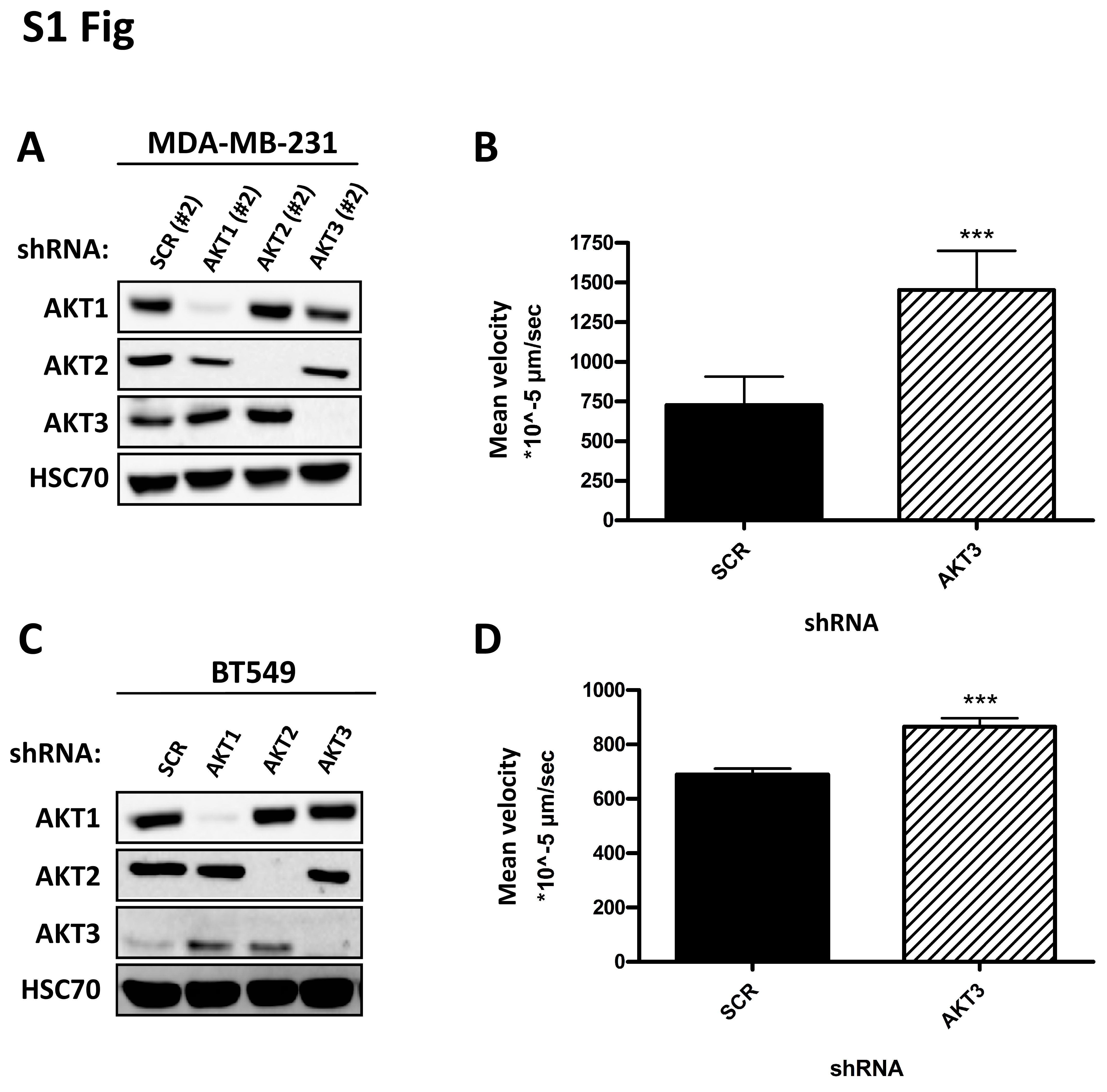

Supplement: S1 Fig — (A) Knockdowns in MDA-MB-231 cells were performed by lentiviral transduction using a second set of independent AKT isoform specific shRNAs (#2). Efficiency was confirmed by Western blot analysis. (B) Analysis of migration of cells lacking AKT3 using scratch assay technique. A confluent monolayer was scratched using a 200μl pipette tip and cell migration was analyzed using time lapse video microscopy. The mean cell velocity of MDA-MB-231 control and AKT3 knockdown cells is shown. (Bars: SD. ***, p < 0,001). (C) AKT isoform specific knockdowns in BT549 cells were generated by lentiviral transduction using AKT isoform specific shRNAs as described in section 3.6. Knockdown efficacy was confirmed by Western blot analysis. (D) Migration of AKT isoform knockdown cells was analyzed by scratch assay and live cell imaging techniques, as described in section 3.6. The mean cell velocity of BT549 control and AKT3 knockdown cells is shown. (Bars: SD. ***, p < 0,001). (TIF) [file pone.0146370.s001.tif]

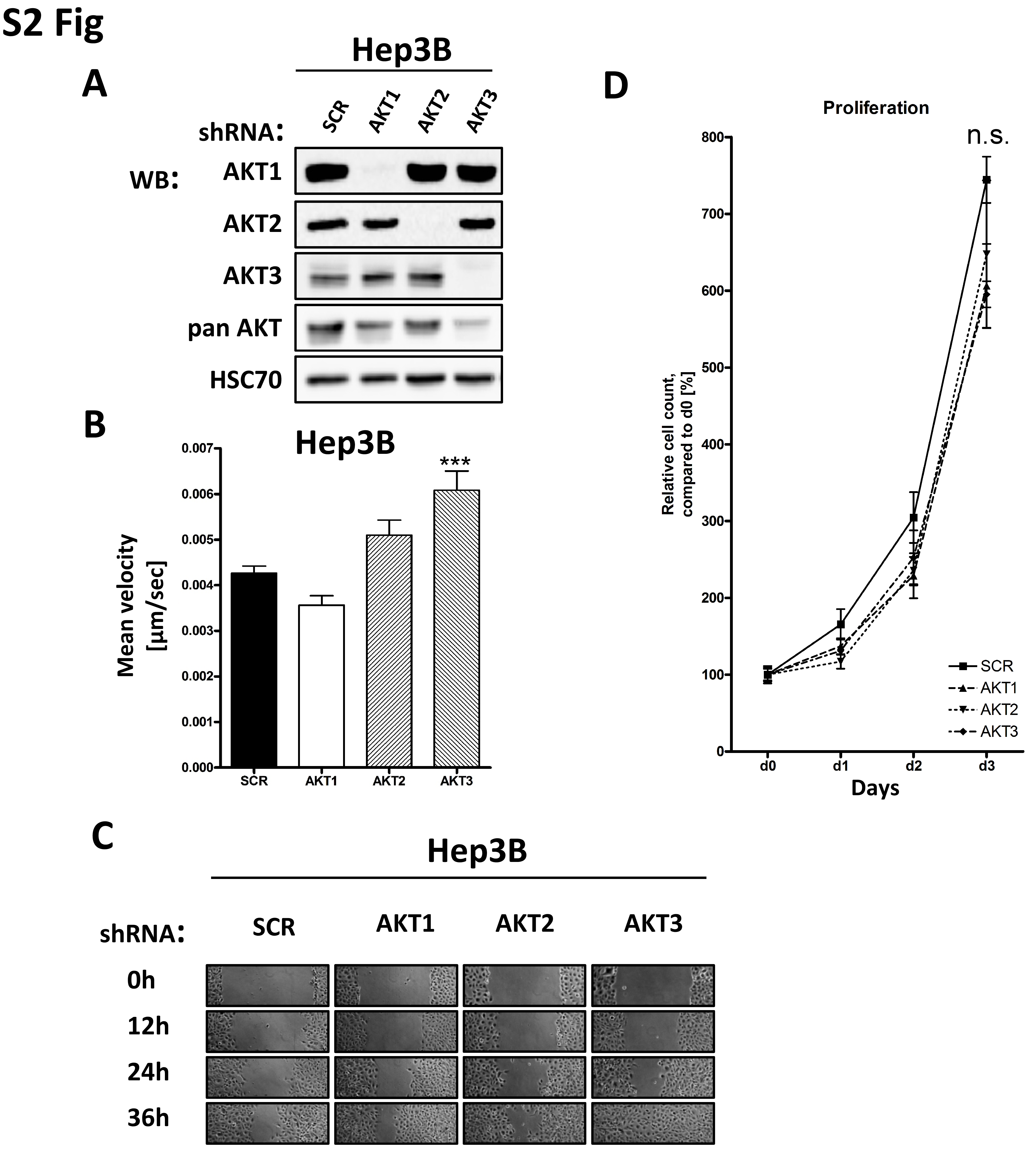

Supplement: S2 Fig — (A) AKT isoform specific knockdowns in Hep3B cells were generated by lentiviral transduction using AKT isoform specific shRNAs. Knockdown efficacy was confirmed by Western blot analysis. (B)-(C) Analysis of cell migration using scratch assay technique. A confluent monolayer was scratched using a 200μl pipette tip and cell migration was analyzed using time lapse video microscopy, as described in section 3.6. Mean single cell velocity of Hep3B control and AKT isoform knockdown cells is given in (B). One representative experiment out of three is shown (Bars: SD. **, p < 0,01. ***, p < 0,001). (C) Representative images of the scratch assay after 0, 12, 24 and 36 hours are shown. (D) Proliferation was analyzed by manual cell counting over four consecutive days, performed in triplicates. Proliferation is shown as relative cell count normalized to day 0 (Bars: SD, n.s., p>0,05). (TIF) [file pone.0146370.s002.tif]
